# Predictors of Suboptimal Response After Radiofrequency Ablation of Benign Thyroid Nodules

**DOI:** 10.3390/jcm14165719

**Published:** 2025-08-12

**Authors:** Giacomo Di Filippo, Fabio Medas, Giulia Gobbo, Leonardo Rossi, Giovanni Lazzari, Dorin Serbusca, Eleonora Morelli, Federico Cappellacci, Marco Puccini, Gabriele Materazzi, Gian Luigi Canu

**Affiliations:** 1 Endocrine Surgery Unit, Department of Surgery and Oncology, University of Verona, 37134 Verona, Italy; giacomo.difilippo@aovr.veneto.it (G.D.F.); giovanni.lazzari@aovr.veneto.it (G.L.); dorin.serbusca@aovr.veneto.it (D.S.); eleonora.morelli@aovr.veneto.it (E.M.); 2 Department of Surgical Sciences, University of Cagliari, SS554 Bivio Sestu, Monserrato, 09042 Cagliari, Italy; fabiomedas@unica.it (F.M.); fedcapp94@gmail.com (F.C.); gianl.canu@unica.it (G.L.C.); 3 Otolaryngology-Head and Neck Surgery Department, University of Verona, 37134 Verona, Italy; 4 Endocrine Surgery Unit, University Hospital of Pisa, Via Paradisa 2, 56100 Pisa, Italy; rossi.leonardo.phd@gmail.com (L.R.); marco.puccini@unipi.it (M.P.); gmaterazzi@yahoo.com (G.M.)

**Keywords:** RFA, thyroid nodules, volume reduction rate, BMI

## Abstract

**Objective**: Radiofrequency ablation (RFA) has gained recognition as a minimally invasive alternative to surgery for managing symptomatic benign thyroid nodules. However, predicting RFA efficacy remains challenging, especially in identifying patients who may require multiple treatment sessions. The aim of the present study is to identify predictors of suboptimal volume reduction (SVR), defined as failure to achieve a volume reduction greater than 5% between 6 and 12 months after procedure and persistence of symptoms. **Methods**: A retrospective single-center analysis of consecutive patients who underwent a single RFA procedure for benign thyroid at Verona University Hospital between 2020 and 2023 was conducted. Clinical data, including nodule volume and compressive symptoms, were collected preoperatively and at 1, 6, and 12 months post-RFA. Regression analysis was performed to identify predictors of SVR and persistence of symptoms. **Results**: A total of 50 patients were included. Baseline nodule volume, higher ACR-TIRADS score, and higher body mass index (BMI) were identified as independent predictors of SVR (*p* < 0.05). At 12 months, 18.4% of patients reported persistent compressive symptoms; however, no significant predictors of symptom persistence were identified. **Conclusions**: RFA is effective in reducing benign thyroid nodule volume, with a minority of patients experiencing persistent symptoms. Baseline nodule volume, ultrasonographic characteristics, and BMI significantly influence RFA outcomes, suggesting the need for additional procedures. Further studies are required to identify predictors of treatment response, enhancing patient selection and optimizing therapeutic efficacy.

## 1. Introduction

Thyroid nodules are among the most common endocrinological conditions, affecting up to 65% of the general population, with a higher prevalence in women [1]. The incidence of thyroid nodules in the general population has risen significantly in recent years, also due to the widespread use of ultrasound as the primary method for evaluating the thyroid gland [2]. Most thyroid nodules are benign and may benefit from nonsurgical management. Asymptomatic nodules in euthyroid patients can be followed over time, since they are characterized by a slow growth pattern and often undergo size stabilization in the post-menopausal age [2,3]. However, a minority of benign nodules may cause compressive symptoms due to their mass effect on the trachea and the esophagus, determining dysphagia, hoarseness, tightness, dyspnea, and coughing. Large thyroid nodules can also lead to cosmetic concerns. In a minority of cases, thyroid nodules are autonomously functioning, leading to clinical symptoms due to the excessive production of thyroid hormones [4].

Non-surgical and minimally invasive techniques represent a valid alternative to surgical treatment for benign thyroid nodules. These techniques include chemical ablation with ethanol (EA) or alcoholization, and thermal ablation through laser (LTA), radiofrequency (RFA), microwave (MWA), or high-intensity focused ultrasound (HIFU). Regarding thermal ablation, LTA and RFA are considered first-line therapies, while MWA is currently undergoing clinical trials to assess its safety and efficacy [5]. To date, the use of RFA is indicated for the treatment of symptomatic patients or patients with cosmetic concerns caused by a benign nodular thyroid pathology [6]. RFA may also play a role in the treatment of autonomous hyperfunctioning thyroid nodules causing thyrotoxicosis [7,8]. Although RFA has proven efficient in achieving nodule volume reduction [9], some patients may require multiple sessions to achieve satisfactory cosmetic, volumetric, or clinical results [10]. Our study aimed to determine early predictors of reduced RFA efficacy to timely identify patients who may require multiple sessions to achieve an adequate volume reduction rate (VRR).

## 2. Materials and Methods

### 2.1. Study Design and Setting

We conducted a retrospective single-center analysis on consecutive patients who underwent RFA for benign thyroid nodules confirmed by two fine needle aspiration cytologies (FNAC) from 1 January 2020 to 31 January 2023 at the University Hospital of Verona and who subsequently underwent an ultrasound follow-up for at least 12 months. Evaluation and management of patients with thyroid nodules were based on ultrasonographic features (via ACR-TIRADS risk stratification tool) [11] and FNAC, as per current guidelines [12].

Exclusion criteria for the RFA procedure were indeterminate or malignant nodules, TIRADS 5 nodules and retrosternal nodules, non-compliant patients, or the patient’s refusal.

Written informed consent for the use of clinical data for research purposes was obtained from all study participants. Study procedures were conducted in accordance with the Declaration of Helsinki.

### 2.2. Data Collection

Clinical data were retrieved from the patients’ computerized medical charts. Clinical data collected included the nodule volume (measured preoperatively and at 1, 6, and 12 months post-RFA by the same operator), the presence of patient-reported compressive symptoms affecting the aerodigestive pathway, and their evolution at 1, 6, and 12 months. VRR was defined as the percentage volume reduction from baseline volume at each time point. RFA efficacy was defined as a VRR of at least 50% at 12 months post-procedure. Suboptimal Volume Reduction (SVR) was defined as failure to achieve a further volume reduction greater than 5% between the 6th and 12th month post-procedure. Specifically, SVR was calculated using the following formula: SVR = (6th month volume − 12th month volume)/6th month volume. This endpoint was meant to include two clinically relevant scenarios: (1) patients whose nodules initially shrank during the first 6 months post-RFA but showed minimal or no additional reduction thereafter, and (2) patients whose nodule volume increased again during the 6–12 month period, indicating regrowth (i.e., negative values of volume reduction).

The 5% cutoff was chosen to account for intra-observer variability in ultrasound-based volume measurements and reflects a conservative threshold for meaningful change. According to SVR, patients were divided into 2 groups: Group A, consisting of patients without SVR, and Group B, consisting of patients with SVR.

### 2.3. RFA Procedure

As per our internal protocol, all RFA candidates underwent routine blood tests, including serum thyroid-stimulating hormone (TSH) and calcitonin, chest X-ray, electrocardiogram (ECG), fiberoptic laryngoscopy, and two FNACs of the benign nodule. The procedure was performed through a trans-isthmic approach, after skin infiltration with local anesthetic (90% mepivacaine solution and 10% bicarbonate) for analgesic and hydrodissection purposes. The moving shot technique was used for the execution of the procedure [13]. After RFA, patients were followed up both clinically and radiologically (i.e., neck US) at 1, 6, and 12 months to assess volume reduction of the nodule and related compressive symptoms. Every patient who achieved SVR was offered additional RFA sessions, which were performed within 18 months after the first procedure.

### 2.4. Statistical Analysis

Continuous variables were described as median and interquartile range (IQR), while discrete variables were described as absolute number and relative percentage. The Chi-square test was used to identify statistically significant differences between groups for discrete variables, while the Mann–Whitney test was used for continuous variables. Univariate and multivariate logistic regression analyses were performed to identify predictors of SVR and of symptom persistence at 12 months. Predictors that were found to be significant at the univariate analysis were subsequently included in a multivariate regression model along with clinically significant parameters to identify independent predictors of the outcomes considered. A receiving operator characteristics analysis was used to identify the optimal preprocedural nodule volume cutoff that predicts SVR, and the Area Under Curve (AUC) was calculated. A value of *p* < 0.05 was considered statistically significant. The analysis was conducted using SPSS software version 25 (IBM Corp., Armonk, NY, USA).

## 3. Results

The study population included 50 patients (74% females), with a median age of 51 years. The median body mass index (BMI) was 25.29 kg/m^2^. Socio-demographic and clinical data of patients included in the study are presented in Table 1.

Forty percent of the patients had a family history of thyroid disease. Twenty-two patients received a diagnosis of multinodular goiter, 25 of single thyroid nodules, two of Plummer’s adenoma, and one of a previously ablated nodule. With regard to the US nodule pattern, 41.3% were solid, 37% presented mixed characteristics, and 21.7% were spongiform. Median volume of the thyroid nodules was 17.85 cm^3^ All patients underwent complete ablation in one session. The RFA procedures lasted a median of 329 s with a median maximum power delivered of 55 W. No peri-procedural complications occurred. Table 2 shows a data comparison between Group A (24 patients) and Group B (26 patients)

**Figure 1:**
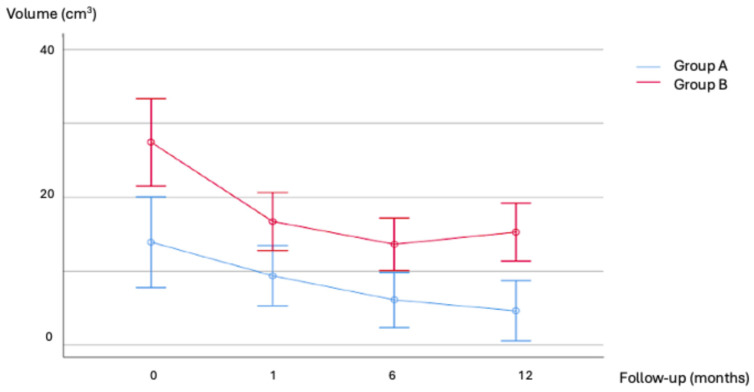
Nodule volume changes in the two subgroups at each time point.

**Figure 2:**
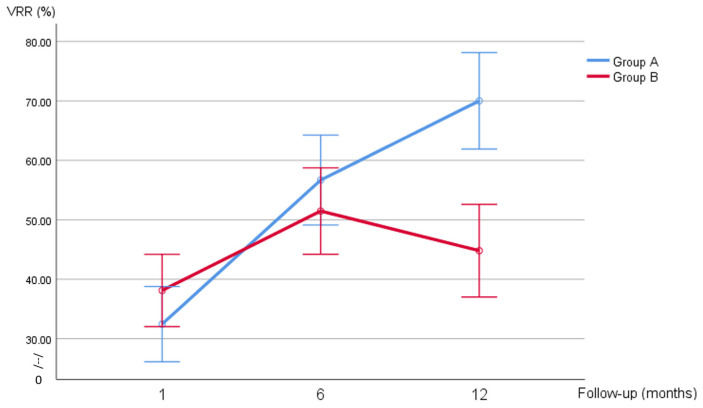
VRR at 1, 6, and 12 months after the procedure divided by group.

**Figure 3:**
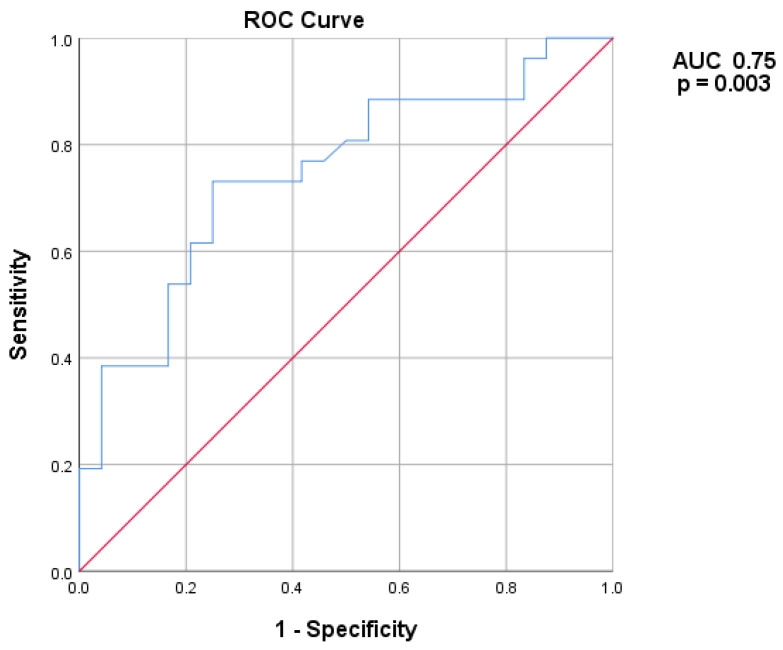
ROC curve detailing the performance of baseline nodule volume in predicting SVR. AUC: area under the curve.

Volume and VRR changes in the two groups at different timepoints are shown in Figure 1 and Figure 2. A preprocedural nodule volume cut-off of 17.85 mL had a sensitivity of 73% and a specificity of 75% in predicting SVR (AUC 0.75, 95% CI 0.61–0.88, *p* = 0.003) (Figure 3). Figure 1 shows the volume changes in the two groups.

At univariate analysis, BMI, male gender, and baseline nodule volume were significant predictors of SVR. Multivariate logistic regression analysis showed that baseline nodule volume, TIRADS > 2, and BMI were independent predictors of SVR (OR 6.5, 1.3, 1.1, respectively, *p* < 0.05) (Table 3).

**Figure 4:**
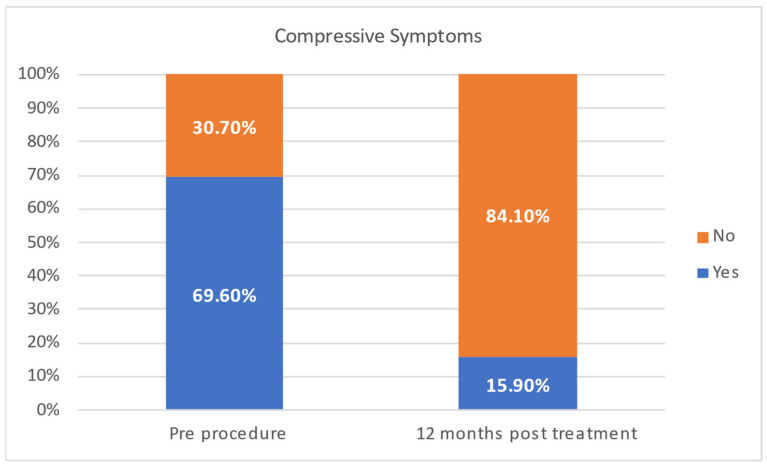
Comparison of compressive symptoms pre- and 12 months post-treatment.

Twenty-five patients (62.5%) presented compressive symptoms. Eleven patients (22%) showed radiographic evidence of tracheal deviation, attributable to the thyroid nodule. Only 18.4% of patients reported persistent compressive symptoms at 12 months (Figure 4). No predictors of compressive symptoms persistence were found at univariate logistic regression analysis. In particular, neither baseline volume nor the VRR at 6 months was found to be a predictor of persistence of compressive symptoms at 12 months.

## 4. Discussion

**Figure 5:**
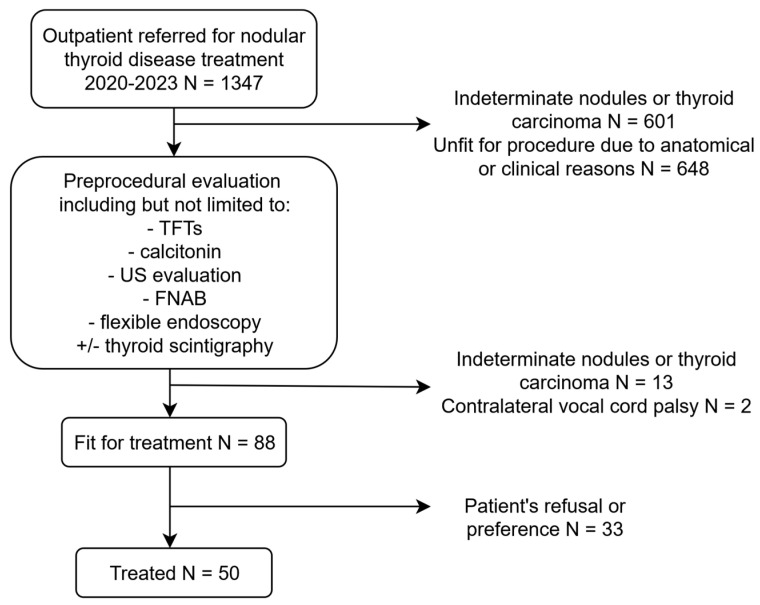
Flowchart of our institution’s outpatients referral for the years 2020 to 2023. TFTs: thyroid function tests; FNAB: fine needle aspiration biopsy.

Radiofrequency thermal ablation of benign thyroid nodules aims to achieve a reduction in nodule volume and a parallel regression of compressive symptoms and cosmetic issues [14]. Some patients demonstrate a less favorable response than others, thus requiring multiple procedures to achieve adequate volume reductions. In our study population, we found that up to 50% of ablated nodules showed SVR, with BMI, nodule baseline volume, and TI-RADS > 2 score identified as independent predictors of reduced RFA efficacy. RFA has been demonstrated to be highly effective in treating thyroid nodules [15]. Negro et al. found that 85.6% of patients treated with a single procedure achieved >50% 1-year VRR [16]. Additionally, Lim et al. found that 13.5% required additional procedures to achieve adequate volume reduction [17]. In our population, more than 50% of patients achieved SVR at 1 year. This may be due to the high median basal volume, the fact that most patients were treated as a second option after surgical consultation, and the heterogeneous indications, including autonomously functioning nodules and multinodular goiter (Figure 5). While most patients may benefit from a single procedure, careful consideration must be given to selecting appropriate candidates and identifying those who might need further interventions, ensuring they are well-informed about the expected outcomes. In our analysis, independent predictors of SVR included the patient’s BMI, basal nodule volume, and ultrasound features. Several studies have investigated predictive factors influencing the response to RFA in benign thyroid nodules, while others have focused on identifying predictors of potential regrowth after treatment [1,6,14,18,19,20]. Basal nodule volume is a well-established risk factor for reduced RFA efficacy. Indeed, Bisceglia et al. identified a basal volume smaller than 22.4 mL as predictive of favorable post-ablation outcomes [14]. This may be attributed to the increased difficulty in achieving complete ablation in larger nodules and the heat sink effect. Patients with larger nodules should be informed that multiple procedures may be necessary to achieve satisfactory volume reduction.

Additionally, Negro et al. demonstrated that nodule composition plays a significant role in influencing RFA outcomes [21]. Specifically, spongiform or predominantly cystic nodules are associated with better VRRs [22]. In our analysis, ACR-TIRADS categories 1 and 2 were predictive of a better response to RFA, likely due to the fact that these categories predominantly include spongiform or mixed nodules, corroborating findings in the literature.

To the best of our knowledge, this is the first study to suggest a possible correlation between BMI and SVR. In a study by Bisceglia et al. [14], people who responded better to RFA had a lower neck circumference, indicating that a larger neck size is associated with poorer RFA outcomes. A higher BMI may be hypothesized to contribute to a larger neck circumference, primarily due to an increased accumulation of adipose tissue in the cervical area. It could be speculated that a greater BMI, possibly associated with a larger neck circumference, could result in increased heat dispersion during the procedure, thereby contributing to suboptimal nodule ablation and potentially necessitating additional sessions to achieve satisfactory outcomes. However, this remains a theoretical consideration, as investigating the pathophysiological mechanisms underlying this association was beyond the scope of the present study. Further experimental research is warranted to explore and validate this hypothesis.

At 12 months post-treatment, only 18.4% of patients reported persistent compressive symptoms. Notably, commonly considered factors such as VRR and preprocedural nodule volume [23] did not show a significant association with the persistence of symptoms. The lack of significant predictors of the persistence of compressive symptoms suggests that other patient-related factors may be more influential in determining whether these symptoms persist after ablation. For instance, it can be hypothesized that an underlying thyroiditis may contribute to persistent symptoms due to chronic inflammation, which is not directly alleviated by nodule reduction. Additionally, anatomical factors such as the ratio between neck circumference and thyroid nodule volume may influence the degree of mechanical compression and the patient’s perception of symptoms, regardless of nodule size or volume reduction. However, this remains speculative, as there is currently no direct evidence supporting this association.

The present study was conducted at a single center by a single operator, ensuring consistency in the procedural approach and minimizing inter-operator variability. Moreover, a wide array of variables was collected and analyzed, allowing for a comprehensive assessment of potential factors influencing the outcomes. However, the small sample size and the retrospective nature of the study may reduce the statistical power of the analysis, and its monocentric nature may limit the generalizability of our findings to other settings. Moreover, the absence of a standardized symptom assessment tool may limit the accuracy and objectivity of symptom evaluation following RFA. Further studies are needed to better understand the procedural and patient-related factors contributing to SVR and symptoms’ persistence, as well as to identify reliable predictors that could enable more personalized and effective treatment strategies.

## 5. Conclusions

This study highlights RFA’s capability of achieving significant volume reduction in a subset of patients while identifying potential characteristics that may hinder its efficacy, with only a minority of patients in our study reporting persistent compressive symptoms at 12 months post-treatment. Additionally, alongside baseline nodule volume and ultrasonographic nodule characteristics, higher patients’ BMI has been identified as an independent predictor of poorer response to treatment. No significant predictors for the persistence of compressive symptoms were found. Further prospective studies are warranted to explore patient-specific factors influencing symptom persistence and response to treatment, allowing for the identification of patients who may need additional procedures to achieve optimal outcomes.

## Figures and Tables

**Table 1:** Demographic, clinical, and imaging information of study patients.

Variable		N (%); Median [IQR]
**AGE**		51 (46–60)
**GENDER**	Female	37 (74.00%)
	Male	13 (26.00%)
**WEIGHT (KG)**		67.0 (60–78)
**HEIGHT (M)**		1.65 (1.61–1.70)
**BMI**		25.28 (22.41–26.84)
**ACR-** **TIRADS**	1	11 (23.90%)
	2	12 (26.10%)
	3	18 (39.10%)
	4	5 (10.90%)
	Missing	4
**AP (CM)**		2.50 (1.90–2.96)
**TV (CM)**		3.20 (2.70–3.91)
**SAG (CM)**		4.20 (3.34–4.75)
**VOLUME (CM3)**		17.85 (10.27–27.69)
**PROCEDURE TIME (S)**		329 (236–447)
**MAXIMUM POWER (W)**		55 (55–65)
**DIAGNOSIS**	MNG	22 (44.00%)
	Previously ablated nodule	1 (2.00%)
	Toxic nodule	2 (4.00%)
	Single thyroid nodule	25 (50.00%)
**COMPRESSIVE SYMPTOMS**	Yes	25 (50.00%)
	No	15 (30.00%)
	missing	10 (25.00%)
**TRACHEAL DEVIATION**	Yes	11 (22.00%)
	No	39 (78.00%)
**VRR (%)**	1 month	32.60 (26.16–47.42)
	6 months	54.67 (40.70–68.94)
	1 year	58.24 (48.01–71.88)

IQR: interquartile range; TIRADS: Thyroid Imaging Reporting and Data Systems; BMI: body mass index; AP: anteroposterior; TV: transverse; SAG: sagittal; MNG: multinodular goiter; VRR: volume reduction rate.

**Table 2:** Sociodemographic and clinical data comparison between Groups A and B.

	Group A	Group B	
	N (%), Median [IQR]	N (%), Median [IQR]	*p*-Value
**Age**	50 (42–59)	55 (46–64)	0.18
**Gender**			0.04
**Female**	21 (87.50%)	16 (61.50%)	
**Male**	3 (12.50%)	10 (38.50%)	
**Weight (kg)**	62.50 (59.00–75.00)	69.50 (60.00–84.00)	0.19
**Height (m)**	1.65 (1.62–1.70)	1.66 (1.61–1.70)	0.78
**BMI**	24.61 (21.77–26.71)	25.77 (23.37–29.32)	0.09
**ACR-TIRADS**			0.56
**1**	5 (22.70%)	6 (25.00%)	
**2**	7 (31.80%)	5 (20.80%)	
**3**	8 (36.40%)	10 (41.70%)	
**4**	2 (9.10%)	3 (12.50%)	
**Missing**	4		
**AP (cm)**	2.10 (1.85–2.90)	2.76 (2.35–3.10)	0.02
**TV (cm)**	2.86 (2.29–3.32)	3.84 (2.98–4.21)	0.003
**SAG (cm)**	3.60 (3.12–4.50)	4.40 (4.13–4.92)	0.01
**Volume (mL)**	12.05 (5.71–19.10)	25.08 (5.30–33.00)	0.003
**Procedure time (s)**	319 (242–402)	352 (220–467)	0.46
**Maximum power (W)**	55 (55–63)	55 (55–65)	0.67
**Diagnosis**			0.733
**MNG**	11 (45.80%)	11 (42.30%)	
**Previously ablated nodule**	1 (4.20%)	-	
**Toxic nodule**	1 (4.20%)	1 (3.80%)	
**Single thyroid nodule**	11 (45.80%)	14 (53.80%)	
**Compressive symptoms**			0.283
**No**	7 (30.70%)	8 (47.10%)	
**Yes**	16 (69.60%)	9 (52.90%)	
**Tracheal deviation**			0.119
**No**	21 (87.50%)	18 (69.20%)	
**Yes**	3 (12.50%)	8 (30.80%)	
**VRR (%)**			
**1 month**	31.44 (21.40–38.43)	37.65 (26.98–47.53)	0.151
**6 months**	57.10 (41.56–73.47)	51.71 (39.42–61.54)	
**1 year**	71.81 (59.18–80.71)	49.58 (33.79–57.46)	
**Composition**			0.75
**Mixed, mainly cystic**	0 (0.00%)	1 (4.20%)	
**Mixed, mainly solid**	7 (31.8%)	9 (37.50%)	
**Solid**	10 (45.50%)	9 (37.50%)	
**Spongiform**	5 (22.70%)	5 (20.80%)	
**Missing**	4		
**Margins**			0.98
**Ill-defined**	12 (54.50%)	13 (54.20%)	
**Smooth**	10 (45.50%)	11 (45.80%)	
**Missing**	4		
**Calcifications**			0.34
**Absent**	21 (95.50%)	20 (100.00%)	
**Macrocalcifications**	1 (4.50%)	0 (0.00%)	
**Missing**	8		
**Vascularization**			0.43
**Absent**	1 (4.50%)	0 (0.00%)	
**Mixed**	16 (72.70%)	15 (65.20%)	
**Perinodular**	5 (22.70%)	8 (34.80%)	
**Missing**	5		

OR: odds ratio; IQR: interquartile range; TIRADS: Thyroid Imaging Reporting and Data Systems; BMI: body mass index; AP: anteroposterior; TV: transverse; SAG: sagittal; MNG: multinodular goiter; VRR: volume reduction rate.

**Table 3:** Univariate and multivariate logistic regression analyses for predictors of SVR.

Univariate				Multivariate		
	OR	95% CI	*p*-Value	OR	95% CI	*p*-Value
**TIRADS > 2**	1.418	0.444/4.531	0.555	6.525	1.073/39.674	0.042
**BMI**	1.181	1.006/1.386	0.042	1.391	1.003/1.930	0.048
**Baseline volume** **(cm^3^)**	1.081	1.021/1.145	0.008	1.108	1.025/1.198	0.010
**Gender**	4.375	1.032/18.556	0.045	1.560	0.198/12.317	0.673

OR: odds ratio; CI: confidence interval; TIRADS: Thyroid Imaging Reporting and Data Systems; BMI: body mass index.

## Data Availability

Data are contained within the article.
